# Increasing influence of heat stress on French maize yields from the 1960s to the 2030s

**DOI:** 10.1111/gcb.12069

**Published:** 2012-12-24

**Authors:** Ed Hawkins, Thomas E Fricker, Andrew J Challinor, Christopher A T Ferro, Chun Kit Ho, Tom M Osborne

**Affiliations:** * NCAS-Climate, Department of Meteorology, University of ReadingReading, UK; † College of Engineering, Mathematics and Physical Sciences, University of ExeterExeter, UK; ‡ Institute for Climate and Atmospheric Science, University of LeedsLeeds, UK; § NCAS-Climate, University of ExeterExeter, UK

**Keywords:** calibration, climate, France, maize, projections, yield

## Abstract

Improved crop yield forecasts could enable more effective adaptation to climate variability and change. Here, we explore how to combine historical observations of crop yields and weather with climate model simulations to produce crop yield projections for decision relevant timescales. Firstly, the effects on historical crop yields of improved technology, precipitation and daily maximum temperatures are modelled empirically, accounting for a nonlinear technology trend and interactions between temperature and precipitation, and applied specifically for a case study of maize in France. The relative importance of precipitation variability for maize yields in France has decreased significantly since the 1960s, likely due to increased irrigation. In addition, heat stress is found to be as important for yield as precipitation since around 2000. A significant reduction in maize yield is found for each day with a maximum temperature above 32 °C, in broad agreement with previous estimates. The recent increase in such hot days has likely contributed to the observed yield stagnation. Furthermore, a general method for producing near-term crop yield projections, based on climate model simulations, is developed and utilized. We use projections of future daily maximum temperatures to assess the likely change in yields due to variations in climate. Importantly, we calibrate the climate model projections using observed data to ensure both reliable temperature mean and daily variability characteristics, and demonstrate that these methods work using retrospective predictions. We conclude that, to offset the projected increased daily maximum temperatures over France, improved technology will need to increase base level yields by 12% to be confident about maintaining current levels of yield for the period 2016–2035; the current rate of yield technology increase is not sufficient to meet this target.

## Introduction

The yield of most crops has increased over the past several decades. However, in the most recent decade, yields have stagnated for many crops in several regions, whereas temperatures have generally increased. The reasons for this stagnation are debated, and could include agricultural policy (Finger, 2010), fundamental genetic limits (Calderini & Slafer, 1998), climate (Lobell & Asner, 2003; Brisson *et al*., 2010), agronomic practice and crop management (Brisson *et al*., 2010). Here, we explore the relative importance of different climatic factors.

Crops are known to be sensitive to various aspects of climate. Persistently elevated temperatures have long been known to accelerate progress towards maturity, and more recently have been shown to have a significant impact on leaf ageing (or senescence; Asseng *et al*., 2011; Lobell *et al*., 2012). Crop responses to shorter periods of high temperature, particularly when coincident with flowering, show yields falling dramatically beyond a threshold temperature (Luo, 2011). This mechanism is observed in both controlled environments and field studies (Ferris *et al*., 1998; Wheeler *et al*., 2000). Similar responses to hot days are beginning to be found at the regional scale: maize yields in the United States have been found to decrease sharply when exposed to temperatures over around 29–30 °C, and this effect outweighs any yield increase due to higher temperatures more generally (Schlenker & Roberts, 2009).

Crop yields are also sensitive to precipitation. Quantifying the relative effect of temperature and precipitation variability is important for understanding impacts and developing adaptation options for future climatic changes. Although this relative importance will vary regionally (e.g. Sakurai *et al*., 2011), some generalizations may be possible through an analysis of mechanisms. For regions where irrigation is increasing, for example, it seems likely that the sensitivity of yield to rainfall will be decreasing. More detailed analyses also indicate that in particular environments (Thornton *et al*., 2010) or at the regional scale (Lobell & Burke, 2008), temperature may be a more significant driver of future yields than precipitation. As temperatures are projected to significantly increase over the next few decades due to continuing anthropogenic emissions of greenhouse gases, whereas precipitation changes are far less certain (Meehl *et al*., 2007; Hawkins & Sutton, 2011), this suggests predictability in future crop yields.

To effectively guide adaptation to future changes, perhaps with different crop growing strategies (Rosenzweig & Tubiello, 2007) or selective crop breeding (Cattivelli *et al*., 2008), there are several key questions to consider. Firstly, can the relative effects of improved technology, precipitation variability and increasing temperatures be quantified? If so, what is the relative size of the effects of rainfall and hot temperatures on yields? And, what level of technology development may be required to overcome any impact of future climatic changes on yield?

In this analysis we develop a methodology to address these questions, focussing on one particular crop (maize) and one country (France) as a case study to better understand the technology trend and the influence of climate on crops. France is chosen specifically for this case study because it has experienced recent extremes of climate. In particular, the heatwave in summer 2003 (Schär *et al*., 2004) has previously been linked to a drop in crop yields across Europe (Easterling *et al*., 2007; Battisti & Naylor, 2009; van der Velde *et al*., 2012).

## Materials and methods

The overall approach is to fit an empirical model to historical observations of climate and crop yield to determine the relative importance of technology, heat stress and precipitation. Climate model simulations are used to make calibrated projections of future heat stress, which are then used to produce yield forecasts assuming no technological development and that the present relationships between climate and yield variability apply in the future. Equivalently, this provides an estimate of how much technological development may be required to maintain yields at present levels. Unless otherwise stated, all uncertainties are given as a 5–95% confidence range.

### Observed climate and crop yield data

The relationships between yield and climate are examined using historical daily precipitation and maximum temperatures from the E-OBS data set (Haylock *et al*., 2008), which is available on a 0.5° × 0.5° grid since 1950, and annual maize yield data from FAOSTAT (http://faostat.fao.org/). We choose to focus on national-level yield data (1961–2010) to provide longer time series to examine trends. Regional yield data (for NUTS2 regions) is only available from 1980 to 2007 which does not allow such a long timescale view. However, we briefly compare the analysis on national scales with the regional data in the Supporting Information.

**Figure 1:**
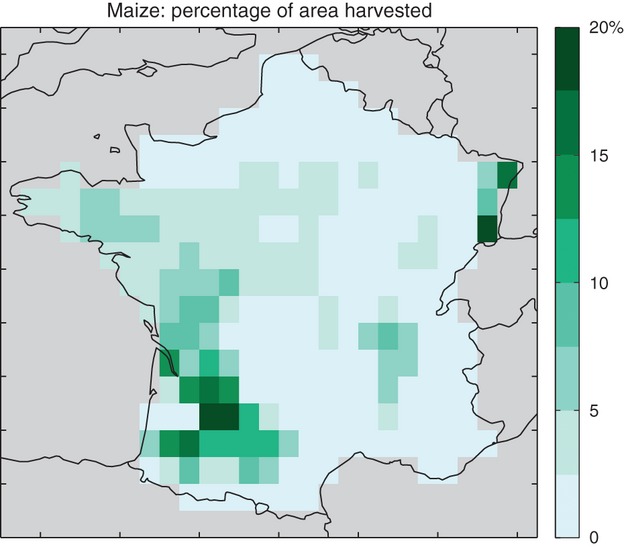
The percentage of land harvested for maize in France in the year 2000, using data from Monfreda *et al*. (2008). The France averages of hot days and precipitation shown throughout the study are weighted using this distribution of maize growth.

We consider two alternatives for measuring heat stress – a simple count of the number of days above a certain critical threshold, and an integrated measure of the degree days above a threshold. Both measures are defined using daily maximum temperature (*T*_max_) during the growing season (June, July and August – JJA), averaged over the whole of France, but weighted by the area of maize harvested in each region (Monfreda *et al*., 2008; Fig. 1). A precipitation index is defined as the mean JJA rainfall, similarly averaged over France. Although the locations of maize growth may have changed over time, similar conclusions are reached if no weighting is applied. In addition, the average planting day may have changed over time (Kucharik, 2006), but given that we are using seasonal averages of climate the effect on our analysis is likely to be small. Finally, we have not considered the details of the timing of the weather events, although this may be extremely important for certain phenological stages of crop growth.

### An empirical model for maize yield

A simple physical understanding for the causes of yield changes suggest that an empirical model for maize yield in France, considering a nonlinear ‘technology’ trend and both the effects of temperature and precipitation, should effectively describe the yield variability. However, the variability in hot days and precipitation is not independent and it is also possible that the effect on yield of an increase in hot days will depend on the precipitation, suggesting that an interaction term may be required (e.g. Runge, 1968; Schlenker & Roberts, 2009). Hence, a generalized additive model (Rigby & Stasinopoulos, 2005) generalized for maize yield (*Y*) is proposed:

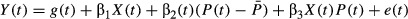
 where *X* and *P* are the temperature and precipitation indices, respectively, 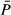 is the mean precipitation index over 1961–2010, the β parameters represent the size of the effects of the various terms, *g*(*t*) is the expected yield in year *t* if there were no hot days and average precipitation and *e* is a stochastic error term. We let *g* be a cubic regression spline to represent the increase in expected yield due to improving technology, which avoids the arbitrary, but often used, assumption that the technology trend is linear with time. The errors are assumed to be normally distributed and temporally independent, but we allow their variance to vary with time to allow for changes in the influence of weather (e.g. precipitation) on yield variability due to technological improvements such as irrigation. To facilitate this we let *e*(*t*) = *h*(*t*) ɛ (*t*) where *h*(*t*) is a cubic regression spline and the ɛ (*t*) are independent standard normal random variables. The unknown β parameters and the spline functions *g* and *h* are all estimated by maximizing a penalized likelihood function (see Supporting Information for more details). Note that β_2_ is time dependent – we assume a similar spline function for its variation. The justifications for the choice of this empirical yield model, as well as tests of simpler and more complex versions, are given below, in Results and in the Supporting Information.

### Empirical yield model selection

Many different empirical models for crop yield have been proposed. A key benefit of choosing a generalized additive model such as Eqn (1) is that all the empirical model parameters, including the nonlinear trend component, are fitted *simultaneously* (e.g. Lobell *et al*., 2011), so as to reduce the chances of overfitting on certain parameters, in contrast to other studies (e.g. Sakurai *et al*., 2011). In addition, the choice of technology trend has been much discussed, with many arbitrary assumptions used. For example, technology trends have been assumed to be linear (e.g. Lobell & Asner, 2003), or quadratic with time (e.g. Schlenker & Roberts, 2009; Lobell *et al*., 2011), or removed using local linear regression (e.g. Sakurai *et al*., 2011) or first differences (e.g. Nicholls, 1997). In some cases the technology trend has not been considered at all (e.g. Tao *et al*., 2006; Knox *et al*., 2012).

Our choice of a cubic spline covers many of these other possibilities as a special case, but is far more flexible. However, to examine the sensitivity to the choice of technology trend in our analysis we considered a version of Eqn (1) with a linear trend for *g*(*t*), rather than a cubic spline. This version of the model produced a significantly poorer cross-validation (see Supporting Information), and we argue that a nonlinear trend is more robust.

In addition, we advocate ‘appropriate complexity’ for an empirical yield model, but additional complexity needs to be considered. For example, the validation statistics of the model were found to be significantly improved if the direct influence of precipitation (β_2_) varies with time (also see e.g. Sakurai *et al*., 2011), and so this factor was included. However, we also tested versions of the empirical model with higher order terms (such as quadratic in *X* and *P*) and also considered time-varying β_1_ and β_3_, but found that these changes did not improve the empirical relationship significantly (see Supporting Information). This yield model also overcomes criticisms of simpler empirical models (e.g. Gregory & Marshall, 2012; Semenov *et al*., 2012) by including an interaction between temperature and precipitation, and basing the choice of possible heat stress indices on the known physical links between hot days and crop growth. Eqn (1) is the simplest version which is found to produce yield estimates which are consistent with the assumptions made in the empirical model, i.e. the residuals are consistent with being independent and random.

### Climate simulations and calibration

Our set of climate model simulations is a QUMP (Quantifying Uncertainty in Model Predictions) ensemble, which consists of 16 variants of the HadCM3 global climate model (GCM) (Gordon *et al*., 2000; Collins *et al*., 2011). This GCM has an atmospheric resolution of 2.5° × 3.75°. Each member of the ensemble differs only in values of particular atmospheric parameters which govern physical processes which are not fully resolved in the model. This ensemble is particularly appropriate for this analysis because it was designed specifically to sample a wide range of climate sensitivities (Collins *et al*., 2011). We use the daily maximum temperature data in JJA during 1960–2035. Historical radiative forcings were used before the year 2000, and the SRES A1B emissions scenario (Nakicenovic, 2000) was followed after 2000.

Here we utilize two approaches for the calibration, both of which are fairly standard in crop modelling, namely ‘bias correction’ (BC) and ‘change factor’ (CF). Both of these methods use historical observations and simulations to derive corrections which can be applied to the future projections, but using different assumptions. In addition, we extend previous methods by also accounting for differences in daily temperature *variability* between the climate simulations and observations, as well as differences in mean climate (Ho, 2010; Ho *et al*., 2012; Hawkins *et al*., 2012), which is particularly important when considering the hot day counts over a threshold (see Supporting Information).

To perform a calibration we require daily *T*_max_ time series from a GCM simulation and observations for the same reference period, which we denote by *T*_REF_(*t*) and *O*_REF_(*t*) respectively. We also need output from the GCM for some future period of the same length as the reference period, *T*_RAW_(*t*). The question remains about how to best combine these three sources of information into the most robust projections of the unknown future observations (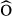
_FUT_) to use as input for crop models. We consider both BC and CF methods, including corrections for the variability as well as the mean climate, to sample this source of uncertainty.

#### Bias correction

The usual BC methodology corrects the projected raw daily GCM output using the differences only in the mean between observations and the GCM in a particular reference period (e.g. Huntingford *et al*., 2005; Ines & Hansen, 2006). However, a more general case when correcting the variability also (Ho *et al*., 2012) is as follows:

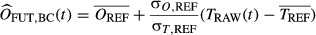
 where σ_T,REF_ and σ_O,REF_ represent the standard deviation of the daily GCM output and observations in the reference period respectively.

#### Change factor

The CF methodology instead utilizes the *observed* daily variability and changes the mean as simulated by the GCM (e.g. Arnell *et al*., 2003; Gosling *et al*., 2009). The general form when correcting the daily variance also (Ho *et al*., 2012) is as follows:

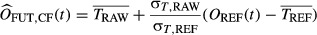
 whereas σ_T,RAW_ represents the standard deviation of the daily raw model output for the future period.

The grid point of the climate model which includes the position of the observations is used in Eqns (2) and 3, so these methodologies also effectively downscale the simulated temperature data to the spatial scale of the available observations. Where the observations are in a location where the climate model has an ocean grid point (grey areas in left column of Fig. 5 later), the nearest land point is selected from the climate model. We use each of the 16 QUMP simulations as independent projections and calibrate *T*_max_ separately for each simulation as above.

The assumptions in the choice of BC or CF are slightly different. If considering future mean climate and no changes in variability, then the two methods produce identical results. However, the more general case above can produce differences in future calibrated climates which are as large as differences between emission scenarios (Ho *et al*., 2012; Hawkins *et al*., 2012). Both methods essentially assume that the change in climate is independent of the mean state, but CF starts from the observations and BC starts from the model output. These methodologies do not consider changing the shape of the distribution of climate data, but this does not matter for a hot days metric in our analysis (see Supporting Information), but may be more important in other situations. Some limited idealized experiments suggested that CF methods may outperform BC methods because they utilize the spatial and temporal variability in the observations, but they may also underestimate the uncertainty because of the limited sampling of the observed variability (Hawkins *et al*., 2012). In the absence of more concrete results, we assume that both methods are equally plausible.

## Results

### Observed changes to yield and climate

**Figure 2:**
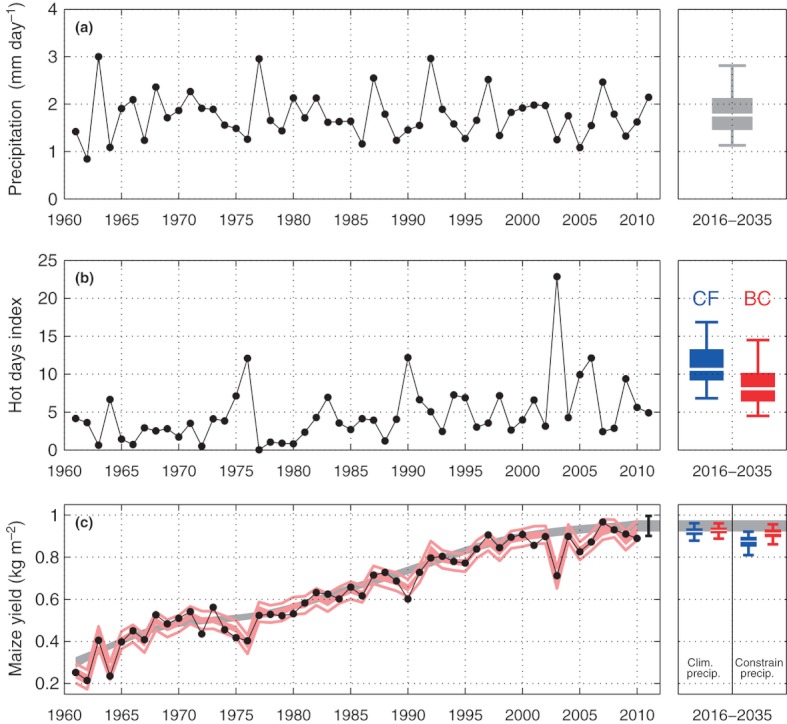
Historical observations and future projections of climate and maize yield for France. (a) Mean JJA precipitation, averaged over all grid cells in France and weighted by the fraction of maize grown. (b) Number of JJA days with *T*_max_ over 32 °C from the E-OBS data set (Haylock *et al*., 2008), averaged over all grid cells in France and weighted by the fraction of maize grown. (c) French maize yields from FAOSTAT (*black points*; http://faostat.fao.org/) and empirical model predictions for the technology trend (*grey shading*) and expected yield (*red shading*) with total uncertainties (*red lines*), using Eqn (1) and considering both temperature and precipitation. The black error bar indicates the forecast for 2011, assuming a flat technology trend since 2010. For the 2016–2035 periods, the boxes show the 25th–75th percentiles and the whiskers indicate the 5th and 95th percentiles. The climatological distribution for JJA precipitation is shown (top), along with the projected and calibrated number of hot days using bias correction (BC) and change factor (CF) methods (middle). The yield projections (bottom) assume a flat technology trend and are shown for both climatological precipitation and precipitation constrained by historical correlations between temperature and precipitation.

Maize is a widely grown crop in France (Fig. 1) and yields have gradually increased from 0.25 kg m^−2^ to a peak of 0.97 kg m^−2^ over the past 40 years^1^ (Fig. 2c). This increase has been attributed to a combination of improved technology (such as fertilizers, pesticides and machinery), more robust and productive crop varieties, as well as CO_2_ fertilization effects (e.g. Gervois *et al*., 2008).

In addition, the number of hot days has increased in France since the 1960s (Fig. 2b, using a 32 °C threshold). Assuming a linear relationship with global mean temperatures suggests a significant increase of 4.5 (0.7–8.3) hot days per 1 °C global temperature rise. Particularly hot years, when compared with nearby years, occurred in 1964, 1976, 1990, 2003 and 2006 (also see Figure S1), and the corresponding maize yield also shows depressed yields in the same years (Fig. 2c). There is no significant trend in precipitation since the 1960s, but variations in maize yields in the 1960s and 1970s seem to be strongly related to precipitation variability (Fig. 2a).

During the heatwave of 2003, the maize yield in France fell to 0.71 kg m^−2^ – a 20% drop on the previous year (also see e.g. van der Velde *et al*., 2010). It seems likely that this yield decrease was related to the hot temperatures that summer – but is this true of less extreme years? And, what is the role of precipitation variability?

### Considering temperature only

**Figure 3:**
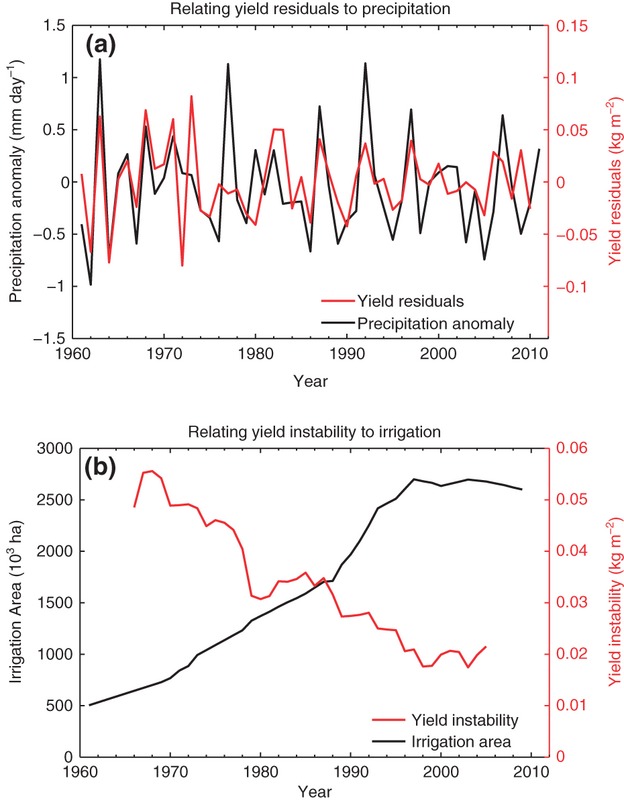
The temperature-only empirical model. (a) The residuals from the expected yield [Eqn 4] as a function of time (red) and precipitation anomaly (black). (b) The running 11-year standard deviation of yield residuals (red), showing a decrease over time, indicating higher yield stability, and the area of irrigation in France from FAOSTAT (black).

To explore these suggestive qualitative links we first utilize a simple form of Eqn (1), considering the effects of temperature alone, i.e. β_2_ = β_3_ = 0. We consider two choices for the temperature index, *X*: firstly, a simple count of the number of days over a critical temperature threshold, and secondly, the integrated temperature–days above a critical threshold. By fitting the suggested yield model [simplified from Eqn 1] to the observed data, it is found that a threshold of 32 °C is the optimal choice for a simple hot day count, and 26.5 °C is optimal for the integrated temperature–days (Figure S3a). For the analysis which follows, we utilize a simple hot day count, which produces a superior fit to the observations than an integrated measure, and also better accounts for differences between the observed and climate model simulated temperature variability (Figure S4). In addition, Fig. 3a (red line) shows the residuals from the expected yield, 

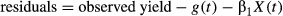
 when considering the count of days above 32 °C as *X*. The running standard deviation of the residuals (red line in Fig. 3b) shows a noticeable decline with time, suggesting increased yield stability recently. In addition, the residuals are well correlated with the mean precipitation anomaly for France (*r* = 0.57, Fig. 3a), especially for the earlier years when there was less irrigation (Fig. 3b). This finding demonstrates the need to include precipitation in the empirical model and is consistent with an increase in irrigation, and/or the development of maize varieties which are more robust to drought, reducing the impact of precipitation anomalies. However, it should be noted that irrigation may also be increased by farmers during periods of high temperature stress, such as the 2003 heatwave, as an adaptation strategy (van der Velde *et al*., 2010).

### Considering both temperature and precipitation

The findings above suggest improving the yield model by adding the effects of precipitation, including an interaction term. However, the influence of precipitation should decrease over time (Fig. 3b), suggesting that β_2_ should be a smooth function, rather than a constant. Note also that the hot day index and precipitation are not independent – the correlation, *r* = −0.46.

Fitting the full model [Eqn 1] to the data retains the finding that a 32 °C threshold is optimal (Figure S3a). When precipitation is included the residuals are consistent with having a constant variance and there is no significant improvement in the yield model by allowing a time-dependent effect for *h*(*t*). For this full yield model, the *h*(*t*) term is therefore assumed to be a constant.

The predicted yield [*Y*; Eqn (1)] with associated uncertainties reliably encompasses the observed yields (Fig. 2c). The red shading indicates the uncertainty in expected yield (without the *e* term) and the red lines indicate the total uncertainty in actual annual yields.

The derived technology trend (*g*) for this yield model increases nonlinearly since 1961 with a noticable plateau in the 1970s (Fig. 2c, grey shading). Although the absolute rate of increase has also slowed again in the most recent decade, the technology trend is still increasing more rapidly than the actual yield. This supports the hypothesis that the recent increase in the number of hot days has caused the actual yield to stagnate (Brisson *et al*., 2010), and is inconsistent with suggestions that the observed plateau in yields is evidence of a fundamental genetic limit on potential yields (Calderini & Slafer, 1998). We now only consider this full empirical yield model.

### Relative importance of temperature and precipitation

**Figure 4:**
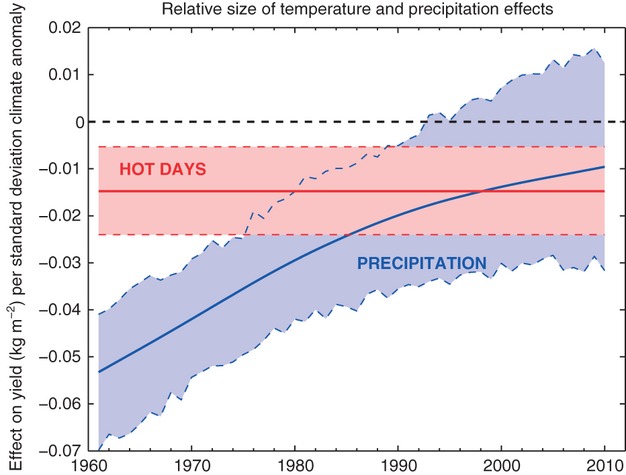
The relative importance of temperature and precipitation for yield, expressed per standard deviation anomaly, assuming median values for the other climate variable, with associated uncertainties. Both effects are shown producing a reduction in yield, i.e. increasing hot days and decreasing precipitation.

A key aspect of this analysis is the ability to determine the relative importance of temperature and precipitation, and how this importance has changed over recent decades. Examining the relative size of the different β parameters suggests that precipitation variability was the dominant contributor to yield variability until around 2000 (Fig. 4). For the most recent decade, the effects of heat stress variability are now as important as precipitation variability, perhaps due to increased irrigation of maize in France (Fig. 3b). Although the interaction term slightly complicates this simple interpretation, it is clear that the *relative* importance of temperature has increased over time. However, it is worth noting that the presence and sign of the interaction term means that hot days become less damaging for yields as precipitation increases.

For the near term (2016–2035), the mean IPCC AR4 projection for summer over Europe is an increase of around 1 °C in mean temperature and a 5% decrease in precipitation from 1980–1999 levels (Meehl *et al*., 2007). However, the uncertainty in precipitation projections is far larger than for temperature (Hawkins & Sutton, 2011), and confidence in the sign of the precipitation change is much lower (Meehl *et al*., 2007), partly because present day simulations of both mean precipitation and its variability are worse than for temperature (Randall *et al*., 2007). In addition, it is likely that temperature will have the largest impact as the projected changes are far further outside the range of natural variability than for precipitation changes (Lobell & Burke, 2008), and because of the seasonal timing of changes in climate (Semenov & Shewry, 2011).

So, for making future projections of crop yields we use the full empirical model considering temperature and precipitation, but focus purely on the effects of changes in temperature, and make the (slightly optimistic) assumption that the climatological distribution of precipitation (from 1961 to 2010) will not change.

### Retrospective calibrated projections of climate

**Figure 5:**
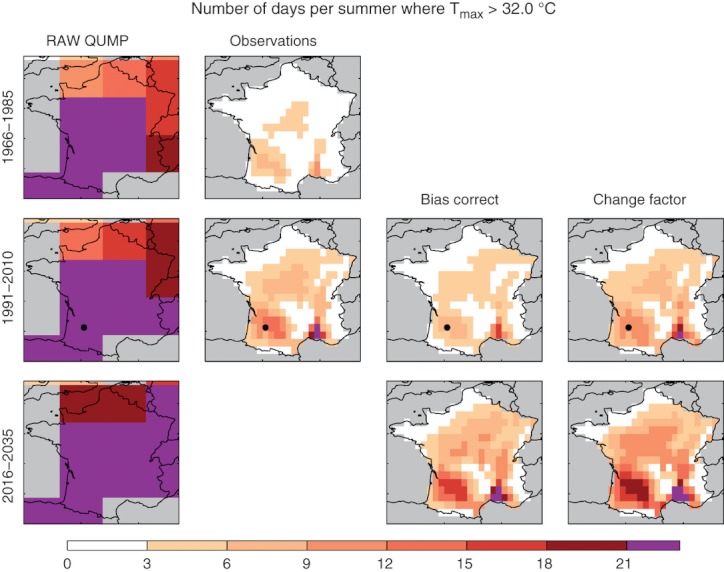
Mean number of hot days in France from the raw QUMP ensemble (left) and E-OBS observations (second column), for various time periods. The mean number of hot days are shown for the out-of-sample prediction of 1991–2010 (second row) and of the future 2016–2035 period (third row) after applying bias correction (BC) calibration (third column) and change factor (CF) calibration (right column), including corrections to daily temperature variability, to each QUMP ensemble member separately.

The construction of the empirical model suggests that yields can be forecast if the number of hot days is known. In principle, climate model simulations can be used to make this projection. However, a key issue in using climate model simulations to study impacts is that the models are biased and do not perfectly reproduce the current climate. For instance, the QUMP ensemble of simulations used here is generally too warm over Europe and produces too many hot days when compared with observations (Fig. 5). Other climate models are less or more biased in this metric (Hawkins *et al*., 2012). Therefore, some calibration is needed before the simulations can be used. To increase confidence in the ability of the calibrated climate model simulations to make forecasts for the number of hot days, we test the predictions retrospectively by comparing with historical observations.

Using the observational data from 1966–1985 only and climate model data from 1966–1985 and 1991–2010, it is possible to make an out-of-sample calibrated projection for the number of hot days observed in the 1991–2010 period using the two different calibration methods (Fig. 5). The calibrations correct much of the warm bias in the raw simulations and produce robust projections of the number of hot days. Note that the variability corrections introduced in Eqns 2 and 3 are essential to producing reliable predictions (compare Fig. 5 with Figure S5).

Averaged over the maize growing regions of France, the raw simulations would produce a hot day index of more than 30 for the 1991–2010 period, but the calibrated projection for the hot day index is 6.3 (3.9–9.4) days (CF) and 4.1 (0.8–7.8) days (BC). The observed hot day index for the 1991–2010 period was 6.4 days (or 5.5 days without the extreme of 2003), an increase on 3.2 days from the 1966–1985 period. The observations are therefore within the uncertainties predicted by the calibrated climate model simulations. It is worth reiterating that we have not used the observations for 1991–2010 to train this climate model prediction – it is made out-of-sample.

**Figure 6:**
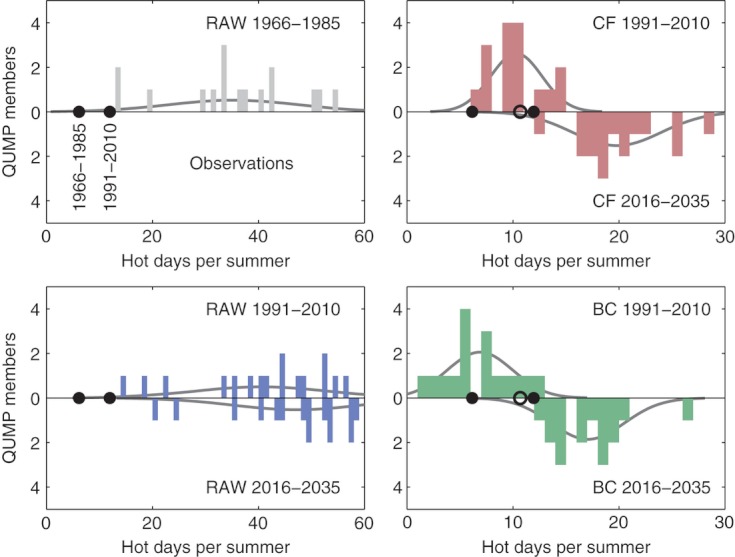
Histograms of 20-year mean projections for the number of hot days for a particular grid point in south-west France (black dot in Fig. 5). Left column: raw QUMP output for reference period (top) and calibrated periods (bottom). Observations are shown in each panel for both reference periods. Right column: Calibrated projections using bias correction (BC, bottom) and change factor (CF, top) for two time periods. The dark grey lines indicate an estimate for a normal distribution from which the QUMP members are drawn. The open black circle indicates the observations for 1991–2010 without 2003. Note the different range on the *x*-axis for the raw and calibrated projections (columns).

When considering a particular location where the largest fraction of the maize is grown (black dots in Fig. 5), the calibrations have narrowed the QUMP spread, reduced the projected number of hot days, and now encompass the observations for both calibration methodologies, unlike the raw simulations (Fig. 6). Histograms are shown for the projections of the QUMP ensemble using raw model output (left column) and calibrated output (right column). The remaining spread in the projected number of hot days represents differences between the climate models used, and also different realizations of climate variability.

When considering projections on annual timescales (Figure S6) it is shown that the retrospectively projected calibrated probability for a 2003-type summer would have been less than 0.6% for the 1991–2010 period. This suggests that the 2003 summer was extreme, even considering the climatic changes, and consistent with other studies examining this heatwave which suggested it was a roughly a 1-in-200 year event (Stott *et al*., 2004).

Overall, these retrospective tests, along with previous studies in idealized situations (Hawkins *et al*., 2012), provide evidence that relatively short lead time (a decade or two) calibrated projections of the number of hot days can be made.

### Projections of future hot days over France

Having demonstrated that the methodology works retrospectively, it can be applied to make a projection for the future period 2016–2035, using 1991–2010 (without 2003) as our training data. There is a projected increase in the number of hot days for many regions (Fig. 5), particularly in regions where maize is grown. Projections for the mean number of hot days per year in the 2016–2035 period for an individual region in south-west France are shown in Fig. 6. The calibrations have narrowed the QUMP range and reduced the projected number of hot days when compared with the raw ensemble. However, the projections still indicate an increase in the number of hot days in 2016–2035 from present, to around 15–20 days per summer for this location. Note particularly that the observations from 2003 are deliberately excluded from the calibration as it was such an extreme year, and could bias the projections to produce too many hot days.

Finally, Fig. 2b shows a calibrated probabilistic near-term projection for the period 2016–2035 for the average number of hot days per year, averaged over France, and weighted for maize growing regions. The projected ranges for 2016–2035 show a likely increase in the number of hot days to around 10 hot days per year, compared with the present day (1991–2010, without 2003) of around 5.5 hot days per year. The two calibration methods do not produce significantly different estimates – 6.8–16.9 (CF) and 4.4–14.4 (BC).

Using annual projections, the chance of a 2003-type summer in the 2016–2035 period is projected to be around 3% per year (Figure S6), equivalent to an increase in risk of about an order of magnitude from the historical period. This suggests that the probability of at least one summer like 2003 is around 50% in this near-term period, assuming independence between years.

### Consequences for future maize yield

At the time of writing, yield data for 2011 has not been published by FAOSTAT. However, the observed climate variability data are available from E-OBS, suggesting a summer close to the long-term mean in terms of precipitation and hot days (Fig. 2). Applying our full empirical model, the yield forecast for 2011 is 0.90–1.00 kg m^−2^, assuming no change in yield due to technology since 2010. Over the past decade yield has increased at roughly 0.005 kg m^−2^ per year due to the technological trend (*g*).

We also define the base level yield (0.92 kg m^−2^) as the mean present day yield (1991–2010, without 2003), corrected for the technology trend increases over the same period. For the future, we do not know the technology trend, and can only make projections for the yield assuming the technology remains constant.

Figure 2c shows probabilistic projections of mean maize yield for France for 2016–2035 using both calibration methodologies (colours) and for two different assumptions on the links between future temperature and precipitation (Figure S7). Assuming future precipitation is independent of temperature, then the projected yield for 2016–2035 is 0.93 (0.89–0.96) kg m^−2^ (BC) and 0.92 (0.88–0.96) kg m^−2^ (CF). However, if the historical correlation between precipitation and temperature is maintained, which we consider more likely, then the predicted yield decreases to 0.91 (0.86–0.96) kg m^−2^ (BC) and 0.88 (0.81–0.92) kg m^−2^ (CF), demonstrating the need to consider correlations between temperature and precipitation in yield projections. We see no reason why a correlation of the same sign would not remain in this near-term period, although its magnitude may change.

Equivalently, according to these climate model simulations and calibration techniques, technology developments must increase yield by 0.11 kg m^−2^ (or around 12% of the current base level yield) to be confident of maintaining yield at present levels. The current rate of yield increase due to technology is not sufficient to meet this target, but would be sufficient to meet the median projection of a required 0.04 kg m^−2^, or a 4% increase in base level yield.

## Discussion

We have quantified the relative importance of temperature and precipitation for historical and future maize yield on France. In addition, we have outlined a methodology for producing calibrated projections of future climate and crop yields, and tested the methods retrospectively. Our main findings are as follows:

1. Our modelled historical technology trend for yield is nonlinear, and suggests a recent slowing in potential yield increases.
2. Maize yield stability in France has increased markedly since the 1960s, likely due to irrigation and technology improvements.
3. The relative importance of precipitation variability for maize yields in France has decreased since the 1960s and the effect of heat stress variability is now as important as precipitation.
4. The number of hot days (above 32 °C), averaged over France, has increased since the 1960s and is projected to increase further to around 10 per summer in the period 2016–2035. For some large maize producing regions, around 15 days per summer are expected.
5. Improved technology will need to increase base level yields by 12% above current levels to be confident about maintaining current maize yields. The current rate of yield increase due to technology is not sufficient to meet this target.
6. Appropriate use of climate model simulations by taking account of differences in both the mean *and* variability of climate is essential, and a rigorous assessment of the characteristics of GCM output is required before its use.

Uncertainty in the projected yields comes from various sources. The component of uncertainty due to the choice of calibration method is not negligible, although CF performs slightly better in retrospective forecasts (see Supporting Information) and idealized modelling studies (Hawkins *et al*., 2012). Each QUMP simulation produces a different calibrated projection, and we have assumed that the QUMP ensemble spans the full range of climate response uncertainty and climate variability for European temperatures. In addition, there are other potential sources of uncertainty in our projections that we have not considered. For example, we have only used a single (SRES A1B) future emissions scenario, but the relative importance of emissions uncertainty is likely to be small for the near term for temperature and precipitation (Hawkins & Sutton, 2009, 2011). The effect of these caveats could be reduced by utilizing the forthcoming CMIP5 climate model simulations which will produce daily data at a higher spatial resolution for more climate models than QUMP (Taylor *et al*., 2012). Finally, we have not considered the effects of changes in ozone, which could be significant for maize yields (e.g. Heagle *et al*., 1972; Hollaway *et al*., 2012). This will be explored in further work.

There has been recent, and we believe correct, criticism of the use of simple empirical relationships between climate and crop yields to infer future yields (e.g. Gregory & Marshall, 2012; Semenov *et al*., 2012). We suggest that the careful consideration of nonlinear technology trends and an interaction between temperature and precipitation is essential in any such empirical model. In addition, the empirical yield model parameters, including the trend component, should be fitted simultaneously.

The availability of smaller spatial-scale crop yield data may also allow improvements in the empirical relationships between hot days, precipitation and yield, although the regional yield time series are not currently long enough to make robust conclusions about long-term trends in the temperature and precipitation effects (see Supporting Information).

Although this is a case study aimed at providing decision-relevant information for a single crop for a single country, future work will aim to provide a wider scale view of future crop yields, based on appropriate use of climate model simulations.

## Footnotes

1 We use SI units for yield, but 1 kg m^−2^ is equivalent to 10 T ha^−1^ or 10^4^ kg ha^−1^

## Supporting Information

The following supporting information may be found in the online version of this article:

**Table S1**Best-fit and uncertainty ranges of parameters [Eqn (1)] for the two different versions of the empirical model, with and without including precipitation. **Table S2.** Validation statistics for maize yield [Eqn (1)] for the different versions of the empirical model, with and without including precipitation, and the full model with a linear *g*(*t*). **Table S3.** Akaike Information Criterion (AIC, Akaike, 1974) values for different versions of the maize yield empirical model [Eqn (1)] with constant or time-varying parameters for β1 and β2. **Figure S1.** The number of days in each summer exceeding 32 °C over France, from the E-OBS v5.0 dataset (Haylock *et al*., 2008). **Figure S2.** The mean summer (JJA) precipitation over France, from the E-OBS v5.0 dataset (Haylock *et al*., 2008). **Figure S3.** Testing the empirical model. **Figure S4.** Quantile-quantile diagnostics for daily *T*_*max*_ for JJA in 1991–2010, for the E-OBS dataset and each QUMP member as labelled, for a particular location (the black dots in Fig. 5). **Figure S5.** As Fig. 5, but without correcting the daily temperature variability. **Figure S6.** Histograms showing number of years across all QUMP members of annual calibrated projections in 20 year periods of the number of hot days for a particular grid point in southwest France (black dot in Fig. 5). **Figure S7.** The relationship between temperature and precipitation and the effect on yield. **Figure S8.** The empirical yield model fitted to two regions of France where a large fraction of the area is harvested for maize (Fig. 1).
