# Supplementary material for: Increasing influence of heat stress on French maize yields from the 1960s to the 2030s
Source: Glob Chang Biol. 2012 Dec 24;19(3):937–47. doi: 10.1111/gcb.12069 (PMC3599478; doi:10.1111/gcb.12069)
Supplement: Supplementary file 1 — Table S1Best-fit and uncertainty ranges of parameters [Eqn (1)] for the two different versions of the empirical model, with and without including precipitation. Table S2. Validation statistics for maize yield [Eqn (1)] for the different versions of the empirical model, with and without including precipitation, and the full model with a linear g(t). Table S3. Akaike Information Criterion (AIC, Akaike, 1974) values for different versions of the maize yield empirical model [Eqn (1)] with constant or time-varying parameters for β1 and β2. Figure S1. The number of days in each summer exceeding 32 °C over France, from the E-OBS v5.0 dataset (Haylock et al., 2008). Figure S2. The mean summer (JJA) precipitation over France, from the E-OBS v5.0 dataset (Haylock et al., 2008). Figure S3. Testing the empirical model. Figure S4. Quantile-quantile diagnostics for daily Tmax for JJA in 1991–2010, for the E-OBS dataset and each QUMP member as labelled, for a particular location (the black dots in Fig. 5). Figure S5. As Fig. 5, but without correcting the daily temperature variability. Figure S6. Histograms showing number of years across all QUMP members of annual calibrated projections in 20 year periods of the number of hot days for a particular grid point in southwest France (black dot in Fig. 5). Figure S7. The relationship between temperature and precipitation and the effect on yield. Figure S8. The empirical yield model fitted to two regions of France where a large fraction of the area is harvested for maize (Fig. 1). [file gcb0019-0937-sd1.pdf]

Increasing influence of heat stress on French maize yields from the 1960s to the 2030s

Ed Hawkins*[1], Thomas E. Fricker[2], Andrew J. Challinor[3], Christopher A. T. Ferro[2,4], Chun Kit Ho[1], and Tom M. Osborne[1]

[1]NCAS-Climate, Department of Meteorology, University of Reading, Reading, UK
[2]College of Engineering, Mathematics and Physical Sciences, University of Exeter, Exeter, UK
[3]Institute for Climate and Atmospheric Science, University of Leeds, Leeds, UK
[4]NCAS-Climate, University of Exeter, Exeter, UK

SUPPLEMENTARY INFORMATION

This supplementary information augments the analysis presented in the main paper published in *Global Change Biology*.

Observed climate

Fig. S1 shows the number of days with a maximum daily temperature ($T_{\max}$) exceeding 32°C over France for each year from 1950 to 2011, according to the E-OBS dataset (Haylock et al., 2008). There is considerable spatial and temporal variability in this quantity, with 1977 and 2003 being two opposite extremes. Fig. S2 shows similar features for mean JJA precipitation, with different extreme years (1962, 1976, 2005).

Validation of the empirical model

Eqn. 1 describes the statistical model for maize yield, using a cubic regression spline for the smooth functions g, h and β_2. The parameters of the model (the β parameters, and the smoothing parameters in g and h) are all estimated by maximising a penalised likelihood function, performed using the gamlss() package (Rigby and Stasinopoulos, 2005) in R (R Development Core Team, 2010). To estimate out-of-sample predictive power we use leave-one-out cross validation (LOOCV) in which we sequentially omit a year from the data, fit the model using the remaining data, and predict the omitted year. The optimal threshold temperature for the variable X (either hot day count or integrated temperature-days) is found by minimising the mean squared error of the LOOCV predictions.

Yield model for temperature alone

Fig. S3a shows the value of the LOOCV RMSE when considering temperature only ($\beta_2 = \beta_3 = 0$), and demonstrates that 32°C is the optimal choice using the hot days count, and 26.5°C is optimal using integrated-temperature days. We choose to use the hot day count for simplicity, and also

*e.hawkins@reading.ac.uk

because we have more confidence that the QUMP simulations can represent this quantity more robustly (see later).

Tables S1, S2 give the best fit parameters, associated confidence ranges and validation statistics for the empirical model. The effect of temperature on yield is summarised by β_1, which suggests a 1% drop in yield from 2010 levels per hot day over 32°C.

We further examine the predictive power of the empirical yield model using a one-step-ahead cross validation method, designed to test the model's ability to predict outside of the range of its fitting data. Here, the response parameter (β_1) is fitted only using years which have *fewer* hot days than the year under test. The fitted parameter is then used to predict the yield response to the hotter year. Fig. S3b demonstrates that the predictions and the estimated uncertainties are robust for predicting the response to out-of-sample years which always have more hot days. Note especially that the yield anomaly in the extreme summer of 2003 is well predicted.

Yield model including precipitation

When considering temperature and precipitation, the optimal choice for the hot days count is again 32°C, and for integrated crop degree days it is 28°C (Fig. S3a). Tables S1, S2 give the best fit parameters, associated confidence ranges and validation statistics for this version of the empirical model. Again, we choose to use the hot day count as it produces a smaller LOOCV RMSE, and also because we have more confidence that the QUMP simulations can represent this quantity more robustly (see later).

As for the yield model for temperature alone, we examine the predictive power of the empirical yield model using a one-step-ahead cross validation method, designed to test the model's ability to predict outside of the range of its fitting data. Here, the response parameters are fitted only using years which have *fewer* hot days (or *less* precipitation) than the year under test. The fitted parameter is then used to predict the yield response to the hotter (or wetter) year. Figs. S3c,d demonstrate that the predictions and the estimated uncertainties are robust for predicting the response to out-of-sample years. Note especially that the yield anomaly in the extreme summer of 2003 is well predicted (Fig. S3c).

Yield model validation - further checks

Note that the version of the empirical model assuming a linear trend has significantly poorer cross-validation statistics (Table S2) than the non-linear version. Finally, the standard version of the empirical model (Eqn. 1) is superior to other versions with constant or time-varying β parameters, according to the Akaike Information Criterion (AIC, Akaike, 1974) (Table S3).

Comparing climate model variability with observations

Fig. S4 shows quantile-quantile plots for the observations and QUMP simulations for JJA $T_{\max}$ in 1991-2010 for a particular grid point in south-west France (black dots in Fig. 5). The thick black lines represent the linear calibration between the QUMP and observational data. The non-linearity seen in the tails of the distributions on both sides indicates that the shape of the temperature distributions are different between the QUMP simulations and the observations. This difference could make calibrated projections of $T_{\max}$ biased.

However, the QUMP simulations are close to the correct distribution up to the 32°C threshold (dashed lines) in all members after linear calibration. As the hot day index simply counts numbers of days over the threshold, and ignores how far above the threshold the temperature is, the

errors in shape of the variability at higher temperatures does not affect this particular metric. Although both an integrated temperature metric and hot day index might be justified physically and empirically (Fig. S3a), the integrated temperature metric would be biased by these differences in shape, so should not be used in this case.

In addition, Fig. S5 is similar to Fig. 5, but does not include a correction for the daily temperature variability in Eqns. 2 & 3, i.e. it uses the standard variants of BC and CF. It can be seen that the BC method in particular does not produce robust estimates of the number of hot days if the variability is not corrected.

Finally, Fig. S6 is an annual version of Fig. 6, showing each individual year in each of the QUMP ensemble members. For bias correction, the shape of the annual distribution does not match the observed distribution particularly well, giving us less confidence in its use for annual values. However, for the 20 year means considered in the paper, the method performs adequately.

These findings demonstrate the need to carefully examine the characteristics of climate variability in any model simulations before using them.

Future yield projections

When producing future projections of maize yield we assume that the present climatological distribution of precipitation (estimated using data from 1961-2010) will not change (Fig. 2). We make two different assumptions about the relationship between temperature and precipitation: first that temperature and precipitation are independent, and second that the observed correlation between precipitation and temperature remains constant in the future period. These cases are illustrated in Fig. S7, which shows the relationship between temperature and precipitation in the observations (white circles), and Monte-Carlo realisations of the future distribution under the first assumption (green) and under the second assumption (blue). The coloured contours represent the yield deficit (which is defined as the difference between the actual yield and the base level of yield due to technology). We see that, under both calibration methods, assuming that the current temperature-precipitation correlation remains constant in the future results in a stronger shift towards large deficits than does assuming independence of temperature and precipitation.

Regional yield data

To examine the potential for using smaller scale yield data we fit our empirical yield model (Eqn. 1) to the yield and climate data from two NUTS2 (Nomenclature of Territorial Units for Statistics) regions in France (Fig. S8) where large fractions of the land is harvested for maize (Fig. 1). The maize yield data is taken from EUROSTAT (http://ec.europa.eu/eurostat/) and is only available from 1980-2007.

The E-OBS weather data (shown in Figs. S1, S2) is averaged over the same NUTS2 regions. Note that the weather can be very different in the two regions. For example, 1990 produced a large number of hot days in the southern region, but not the northern region, and 1992 was very wet in the southern region, but average in the northern region. In 1987 the rainfall anomalies were reversed.

The same empirical model is fit to both sets of regional data (Fig. S8), and produces residuals which are consistent with the model assumptions. However, the shorter time series means that the uncertainties are much larger and robust statements about trends in the effects sizes are not possible. In this instance, the longer temporal information is more important than the smaller spatial information.

References

Akaike, H., 1974: A new look at the statistical model identification. *IEEE Transactions on Automatic Control*, **19**, 716–723, doi:10.1109/TAC.1974.1100705.

Haylock, M. R., N. Hofstra, A. M. G. Klein Tank, E. J. Klok, P. D. Jones, and M. New, 2008: A European daily high-resolution gridded dataset of surface temperature and precipitation. *J. Geophys. Res.*, **113**, D20 119, doi:10.1029/2008JD10201.

R Development Core Team, 2010: *R: A Language and Environment for Statistical Computing*. R Foundation for Statistical Computing, Vienna, Austria, URL `http://www.R-project.org`, ISBN 3-900051-07-0.

Rigby, R. and D. Stasinopoulos, 2005: Generalized additive models for location, scale and shape. *J. Royal Stat. Soc.: Series C*, **54**, 507–554.

Table S1: Best-fit and uncertainty ranges of parameters (Eqn. 1) for the two different versions of the empirical model, with and without including precipitation. The $5 - 95\%$ confidence levels are given in brackets.

	Empirical model version	
Parameter	Temperature only	Temperature and Precipitation
β_1	-0.0101 (-0.0120 to -0.0087)	-0.0277 (-0.0216 to -0.0379)
β_3	0	0.0143 (0.0100 to 0.0210)
h	varying	0.0235 (0.0191 to 0.0280)

Table S2: Validation statistics for maize yield (Eqn. 1) for the different versions of the empirical model, with and without including precipitation, and the full model with a linear $g(t)$.

		Empirical model version	
Statistic	Temperature only	Temperature and Precipitation	Linear $g(t)$
Correlation with trend	0.984	0.993	0.983
Correlation without trend	0.762	0.899	0.819
R^2 with trend	0.969	0.986	0.966
R^2 without trend	0.581	0.841	0.695
LOOCV RMSE [$\mathrm{kg\,m^{-2}}$]	0.050	0.034	0.043

Table S3: Akaike Information Criterion (AIC, Akaike, 1974) values for different versions of the maize yield empirical model (Eqn. 1) with constant or time-varying parameters for β_1 and β_2. More negative values of AIC indicate a superior model. The standard version of the model (Eqn. 1) is shown in bold, and this has the lowest AIC.

β_1	β_2	AIC
Constant	Constant	-196.5
**Constant**	**Time-varying**	**-202.2**
Time-varying	Constant	-195.9
Time-varying	Time-varying	-201.9

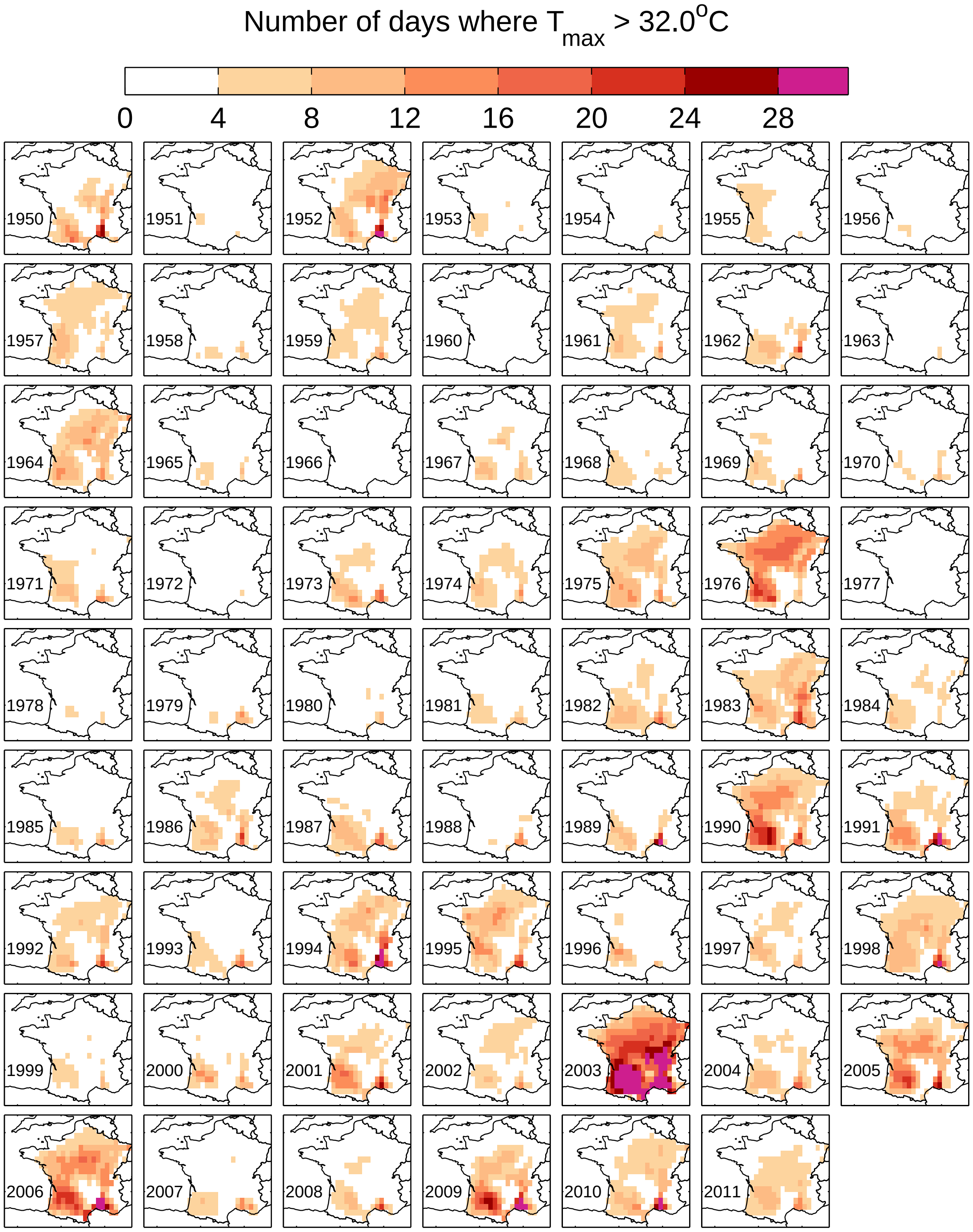

Figure S1: The number of days in each summer exceeding 32°C over France, from the E-OBS v5.0 dataset (Haylock et al., 2008).

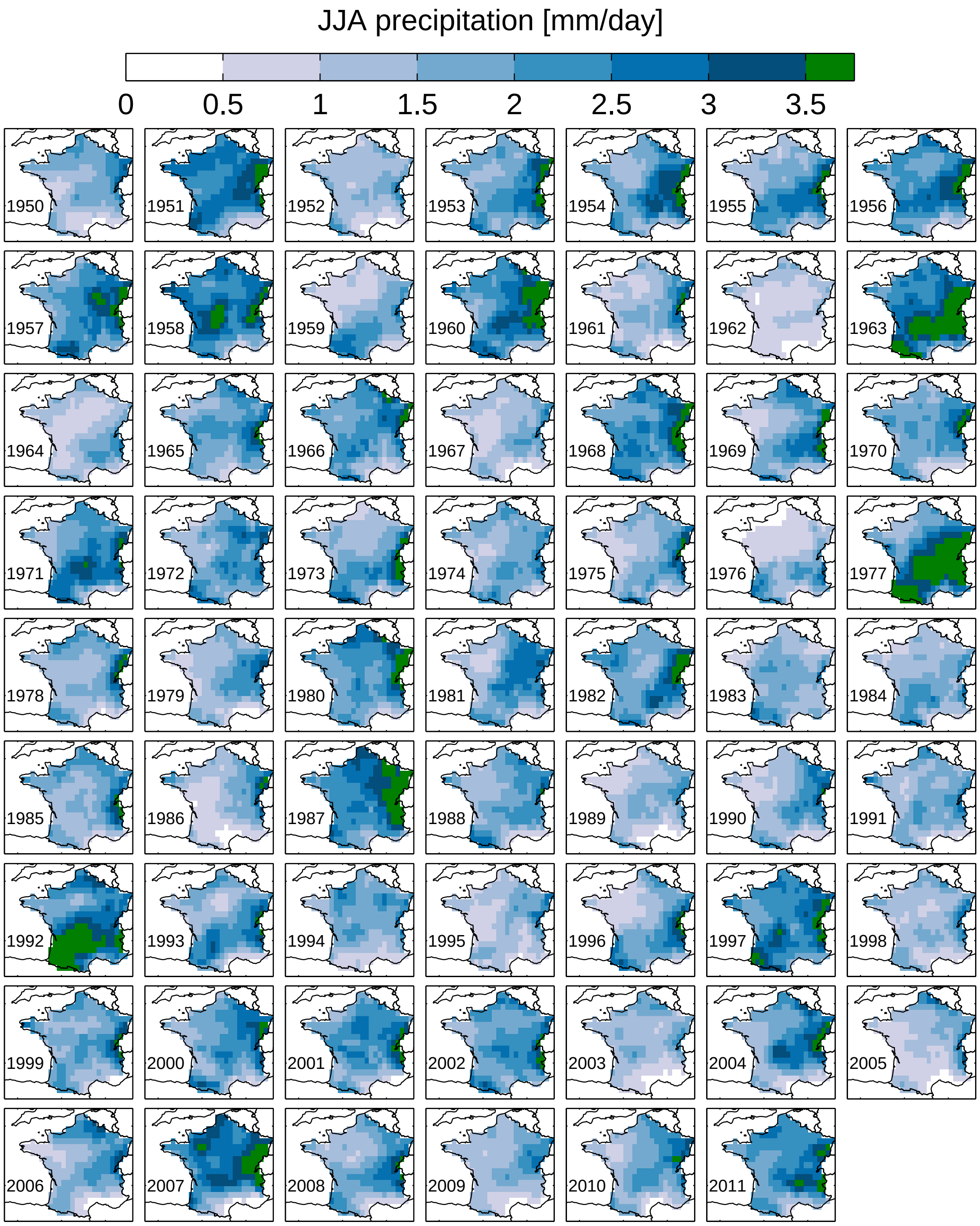

Figure S2: The mean summer (JJA) precipitation over France, from the E-OBS v5.0 dataset (Haylock et al., 2008).

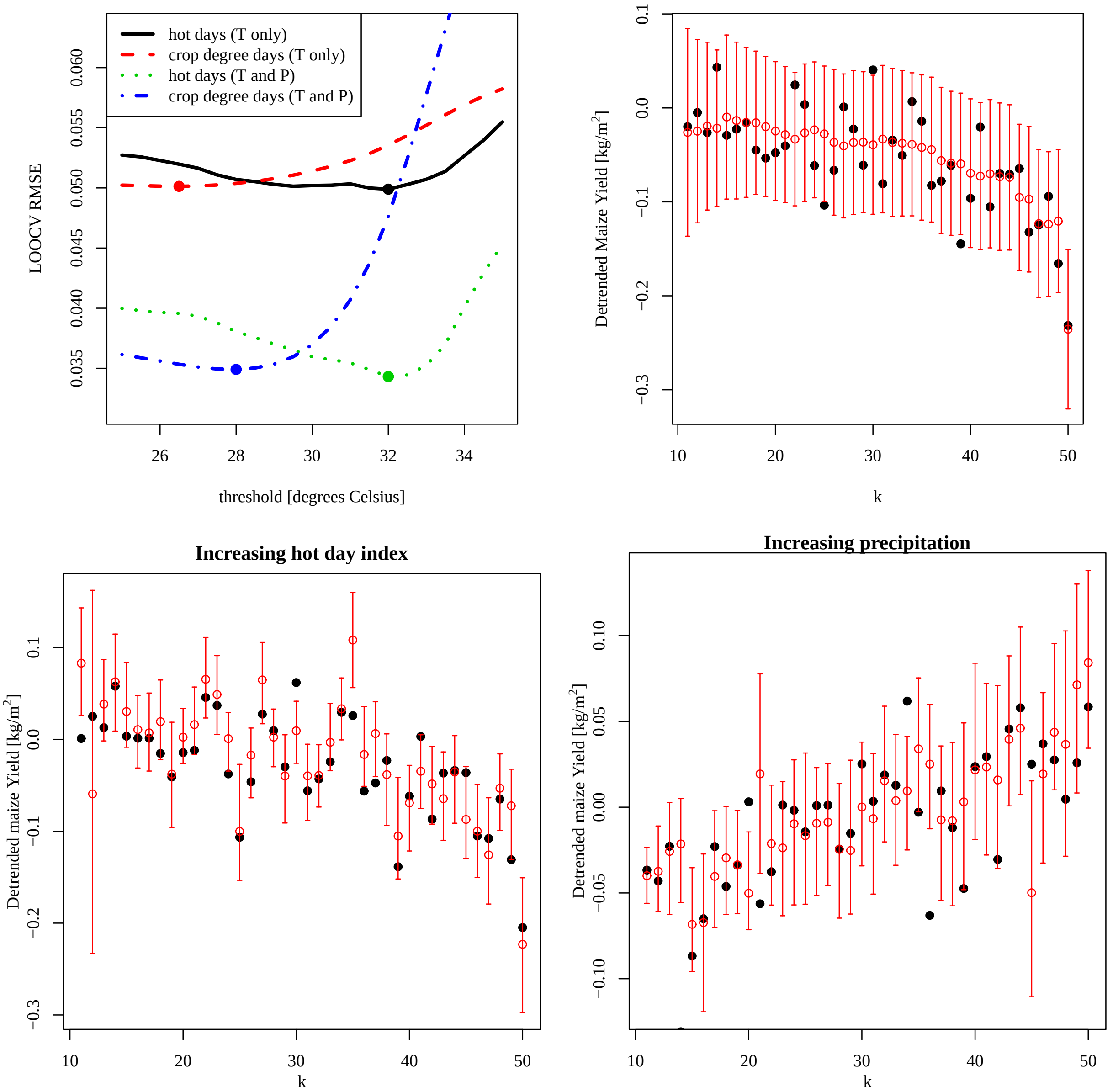

Figure S3: **Testing the empirical model.** (top left) Validation statistics when fitting the empirical crop yield models using leave-one-out cross-validation. (top right) Testing the temperature only empirical model in predicting the response to higher temperatures (one-step-ahead). (bottom) Testing the full empirical model in predicting the response to higher temperatures (left) and precipitation (right). k is an index of years of increasing temperature or precipitation. The black dots are the actual yield, and the red error bars are the out-of-sample predictions of the empirical model.

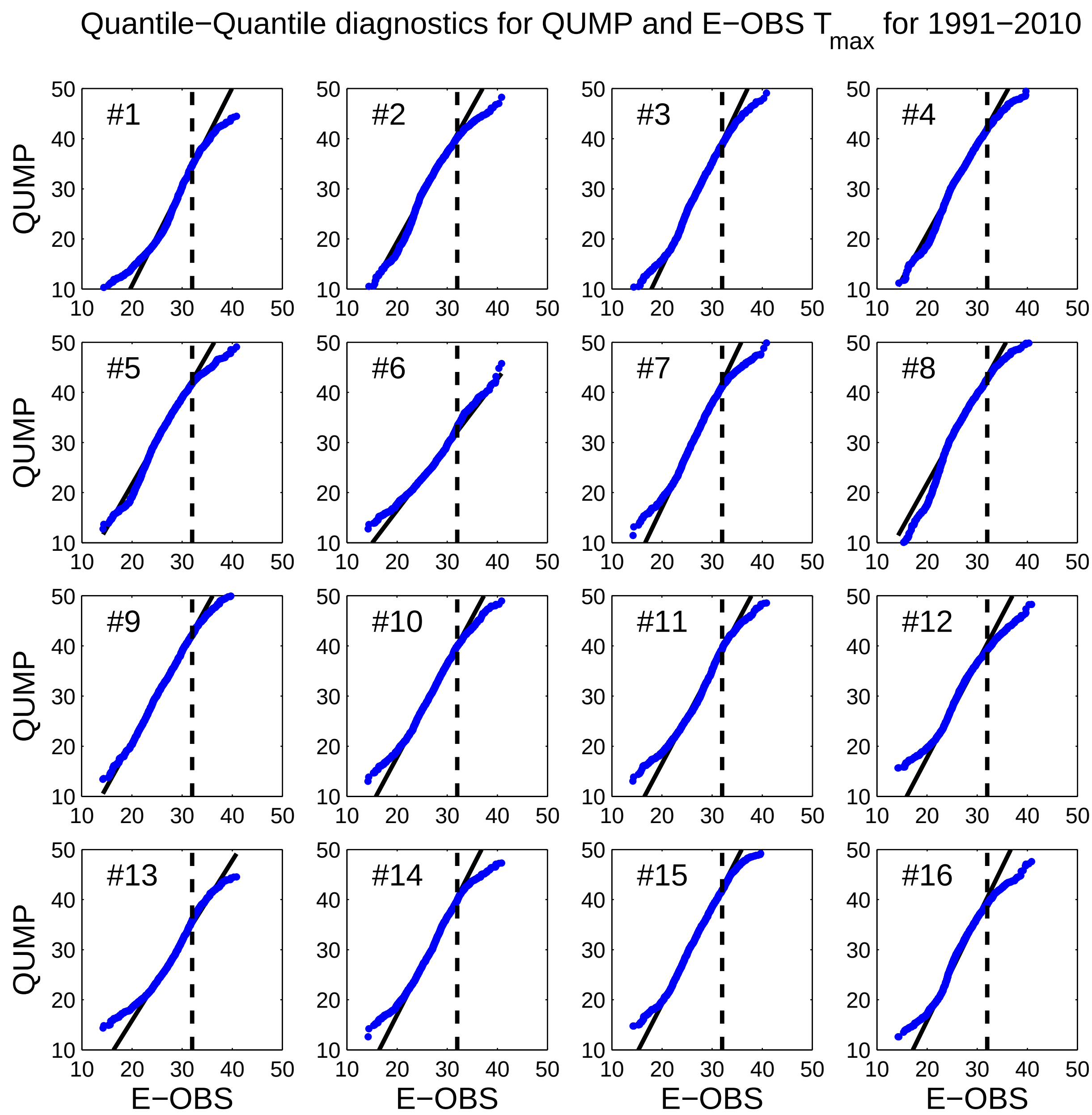

Figure S4: Quantile-quantile diagnostics for daily $T_{\max}$ for JJA in 1991-2010, for the E-OBS dataset and each QUMP member as labelled, for a particular location (the black dots in Fig. 5). The solid black line in all panels represents the linear calibration, and the dashed black line is at 32°C, the threshold for hot days.

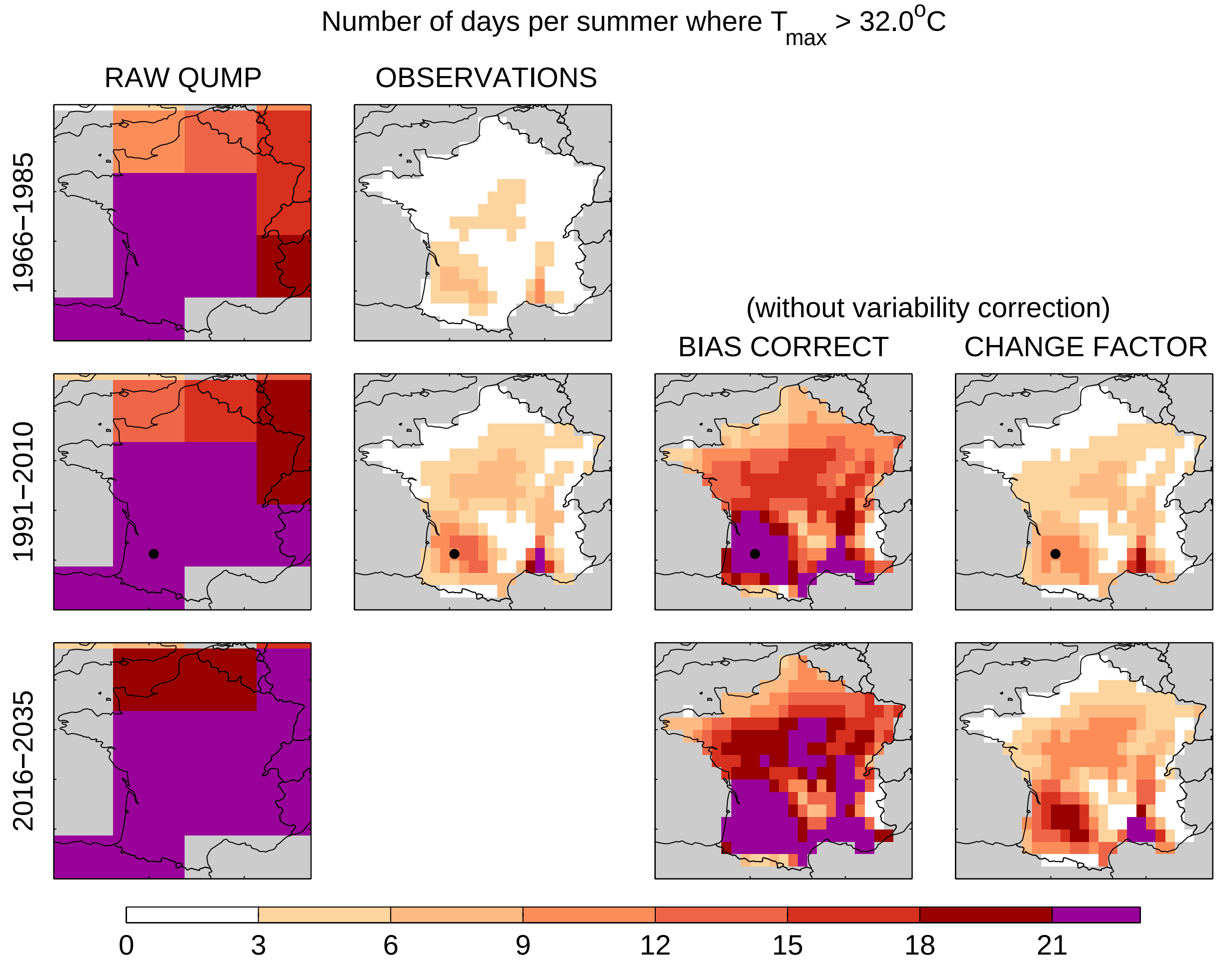

Figure S5: As Fig. 5, but without correcting the daily temperature variability.

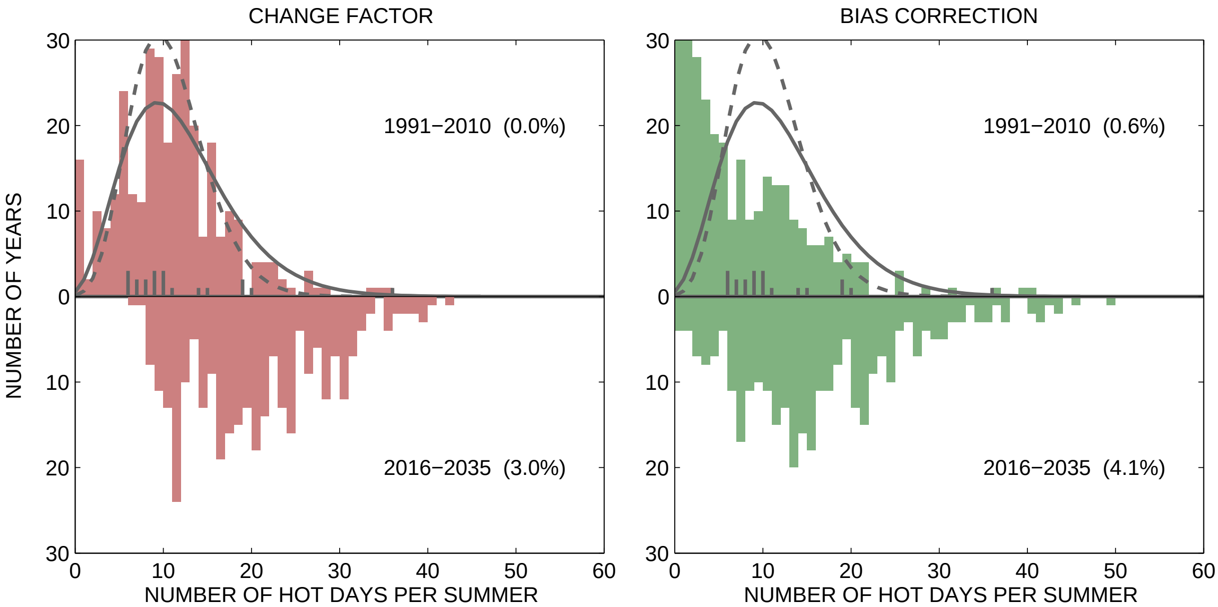

Figure S6: Histograms showing number of years across all QUMP members of annual calibrated projections in 20 year periods of the number of hot days for a particular grid point in south-west France (black dot in Fig. 5). The observations are shown with the grey histogram, and the grey lines show a negative binomial distribution fit to the observations with (solid) and without (dashed) 2003. The labelled percentages indicate the suggested probability of exceeding the number of hot days (44) seen in 2003.

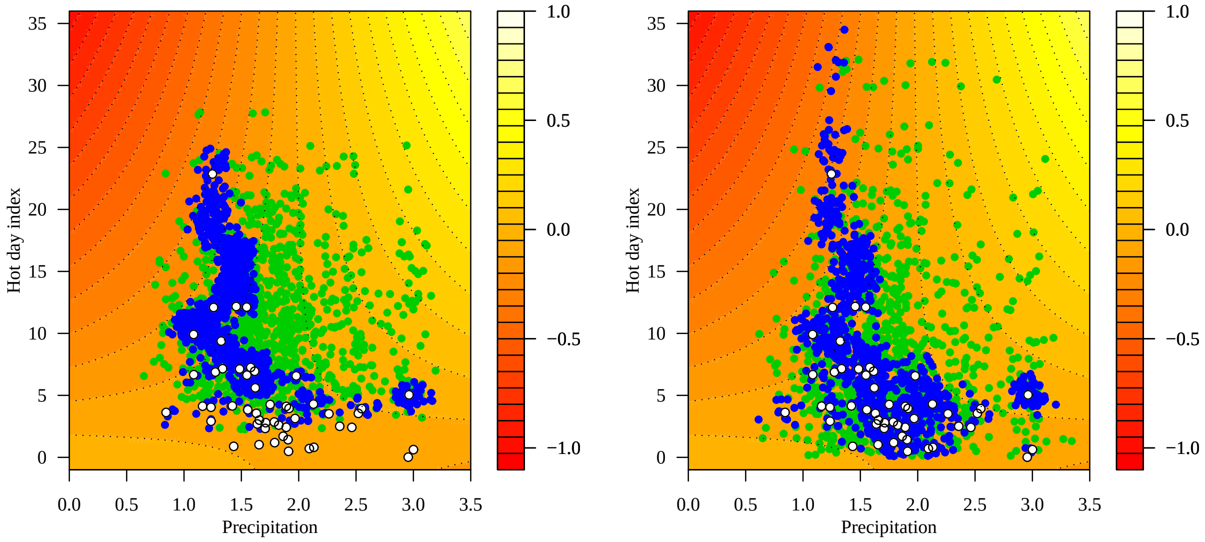

Figure S7: The relationship between temperature and precipitation and the effect on yield. The observations (white circles) are compared with predictions assuming no relationship (green) and constant correlation (blue) for CF (left) and BC (right) calibrations. The filled contours represent the yield deficit [kg m^{-2}].

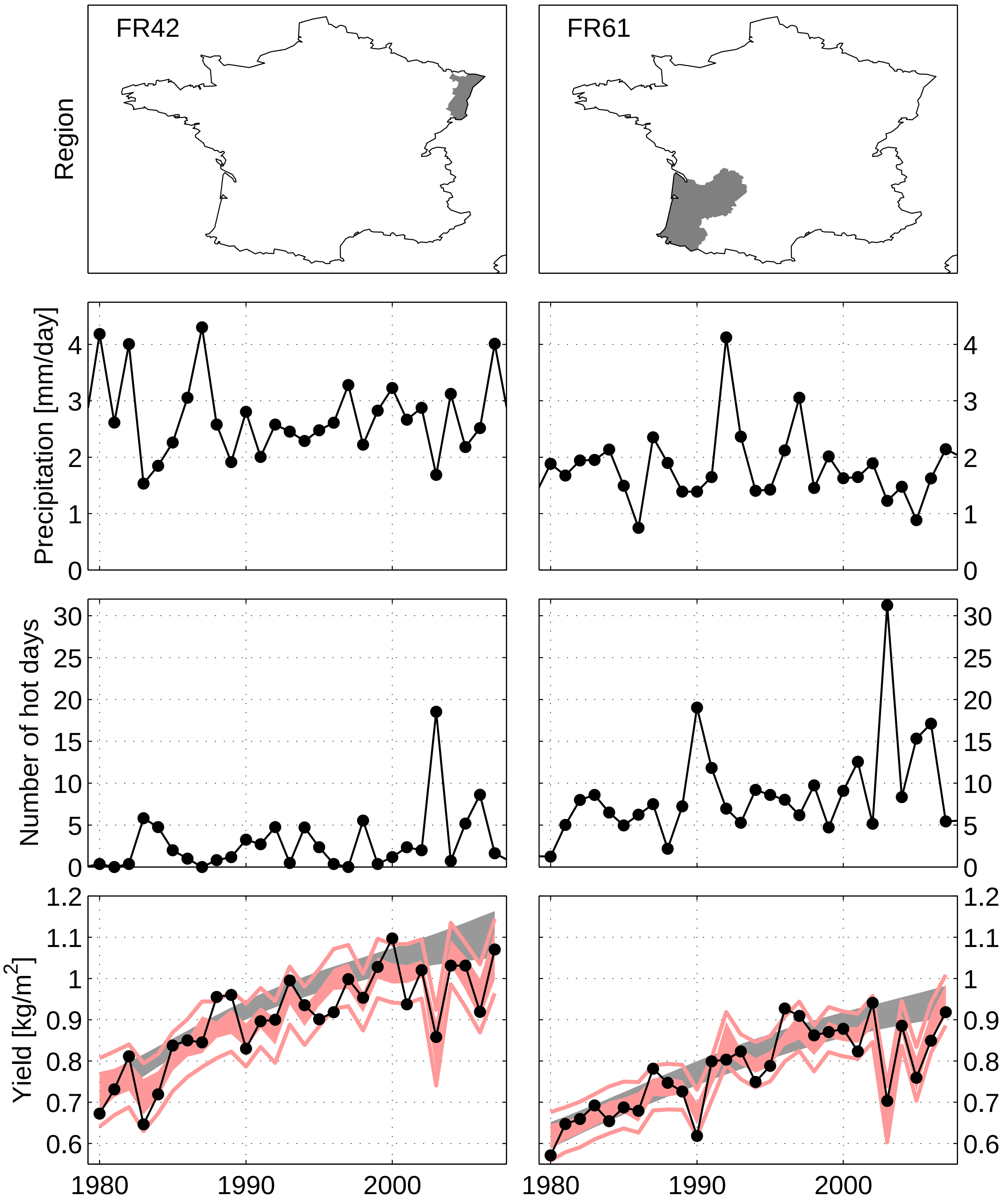

Figure S8: The empirical yield model fitted to two regions of France where a large fraction of the area is harvested for maize (Fig. 1). The maize yield data for these NUTS2 regions is only available from 1980-2007.
